# Association Between Childhood Asthma and Gastroesophageal Reflux Disease in Children: A Systematic Review

**DOI:** 10.7759/cureus.65264

**Published:** 2024-07-24

**Authors:** Nazim F Hamed, Wessal M Alahmad Al Sakran, Ashraf I Serhan, Mohamed Farahat Mohamed Eladwy, Tamer Mohamed Mohamed Elshahhat, Ahmad Salem Abu Lebeh, Sakinah Mohammed Elsharif, Hajar K Alshaqha

**Affiliations:** 1 General Pediatrics, Maternity and Children Hospital, Tabuk, Tabuk, SAU; 2 Pediatrics, Security Force Hospital, Dammam, Dammam, SAU; 3 Pediatrics, Security Forces Hospital Dammam, Dammam, SAU; 4 Pediatric, Security Force Hospital, Dammam, Dammam, SAU

**Keywords:** #childhood asthma, #gastroesophageal reflux disease, #children, #pediatric respiratory health, #gerd

## Abstract

This study aims to comprehensively investigate the association between childhood asthma and gastroesophageal reflux disease (GERD) in children. A thorough search of pertinent databases was done in order to find studies that satisfied the requirements for inclusion. A thorough search of PubMed, Web of Science, SCOPUS, and Science Direct was conducted to find pertinent literature. Twelve studies, including a total of 176,678 patients - 91,447 (51.8%) of them were males - were included in our data. The prevalence of GERD in asthmatic children ranged from 0.7% to 65.3%, with a total prevalence of 3317 (3.6%). The included studies documented that GERD increases the chance of asthma, while asthma raises the risk of GERD. Obesity in asthmatic patients was an independent risk factor for the incidence of GERD. Controlling asthma is significantly impacted by comorbidities like obesity and GRED. The findings of our comprehensive review point to a possible link between juvenile patients with asthma who are referred to secondary and tertiary care facilities and having GERD. Nevertheless, the evidence for this link is weak in a number of situations. Lack of longitudinal research establishing the proper temporal sequence, studies indicating no severity-response relationship, and insufficient data showing a treatment-response relationship all contribute to the uncertainty around the nature and direction of the association. Our findings highlight the need for additional epidemiologic research to investigate the connection between GERD and asthma, including long-term follow-up.

## Introduction and background

Childhood asthma and gastroesophageal reflux disease (GERD) are two common chronic conditions that affect children worldwide. Asthma is a chronic respiratory condition characterized by inflammation of the airways, leading to symptoms such as wheezing, coughing, and difficulty breathing. GERD, on the other hand, is a chronic digestive disorder in which stomach acid flows back into the esophagus, causing symptoms such as heartburn, regurgitation, and chest pain [1].

There is growing evidence to suggest that there may be an association between childhood asthma and GERD in children. Several studies have reported a higher prevalence of GERD in children with asthma compared to those without asthma. Additionally, some studies have found that treating GERD can improve asthma symptoms in children, suggesting a potential link between the two conditions [2].

In this essay, we will explore the association between childhood asthma and GERD in children, including the potential mechanisms underlying this relationship, the impact of GERD on asthma outcomes, and the implications for clinical practice [3].

Several potential mechanisms have been proposed to explain the association between childhood asthma and GERD in children. One possible explanation is that GERD may trigger or exacerbate asthma symptoms through the aspiration of gastric contents into the airways. Stomach acid and other digestive enzymes can irritate the airways, leading to inflammation and bronchoconstriction, which are characteristic features of asthma [4].

Another possible mechanism is that asthma and GERD may share common risk factors, such as obesity, allergies, and genetic predisposition. For example, obesity is a known risk factor for both asthma and GERD, and children who are overweight or obese may be more likely to develop both conditions. Similarly, children with allergies or a family history of asthma may be at increased risk of developing GERD [5].

The presence of GERD in children with asthma has been associated with worse asthma outcomes, including more frequent asthma exacerbations, poorer asthma control, and a lower quality of life. Children with both asthma and GERD may experience more severe asthma symptoms, increased use of asthma medications, and more frequent visits to the emergency department or hospital for asthma-related complications [6].

Additionally, untreated GERD in children with asthma may lead to a poor response to asthma treatment, as acid reflux can worsen airway inflammation and reduce the effectiveness of asthma medications. This can result in uncontrolled asthma symptoms, decreased lung function, and an increased risk of asthma-related complications in children with both conditions [7].

Given the potential impact of GERD on asthma outcomes in children, healthcare providers should consider screening for GERD in children with asthma, especially those who have poorly controlled symptoms or frequent exacerbations. Simple screening tools, such as questionnaires or symptom-based assessments, can help identify children at risk for GERD and guide further evaluation and management [8].

Treatment of GERD in children with asthma may involve lifestyle modifications, such as dietary changes, weight management, and elevation of the head of the bed during sleep. In some cases, medications such as proton pump inhibitors or H2-receptor antagonists may be prescribed to reduce acid reflux and improve the symptoms of GERD. By addressing GERD in children with asthma, healthcare providers can potentially improve asthma outcomes and quality of life for these patients [9].

The rationale behind this study is to address the potential link between childhood asthma and GERD, as understanding this association could lead to improved diagnosis, treatment, and management strategies for both conditions. There is a need to comprehensively evaluate the potential relationship between childhood asthma and GERD in order to better understand the impact of one condition on the other and to potentially identify new avenues for intervention and management. The aim of the study is to systematically review existing literature to determine the association between childhood asthma and GERD in children, with the goal of providing a comprehensive understanding of this potential relationship.

The objectives of the study include conducting a thorough review of relevant literature, analyzing the findings to determine the strength of the association, identifying potential mechanisms or pathways linking the two conditions, and providing insights that could inform clinical practice and future research in this area.

## Review

Methodology

Study Design

The study was a systematic review of existing literature on the association between childhood asthma and GERD in children.

Search Strategy

A comprehensive search was conducted in electronic databases such as PubMed, Scopus, and Web of Science using relevant keywords related to childhood asthma, GERD, and their potential association. Additional sources, such as reference lists of relevant articles and grey literature, were searched.

Inclusion Criteria

Studies included in the review were original research articles that investigate the association between childhood asthma and GERD in children. Studies must be published in English and include participants under the age of 18. Studies conducted after 2010 were included.

Exclusion Criteria

Studies that do not focus on childhood asthma or GERD, review articles, case reports, and studies with insufficient data were excluded from the review.

Data Extraction

Data were extracted from the included studies using a standardized data extraction form. Information collected included study characteristics, participant demographics, study design, measures of association, and key findings related to the association between childhood asthma and GERD.

Quality Assessment

The quality of the included studies was assessed using established tools such as the Newcastle-Ottawa Scale for observational studies [10].

Data Synthesis

Findings from the included studies were synthesized narratively, highlighting key findings related to the association between childhood asthma and GERD.

Reporting

The findings of the systematic review were reported following the Preferred Reporting Items for Systematic Reviews and Meta-Analyses (PRISMA) [11] guidelines to ensure transparency and reproducibility of the study.

Results

Search Results

**Figure 1:**
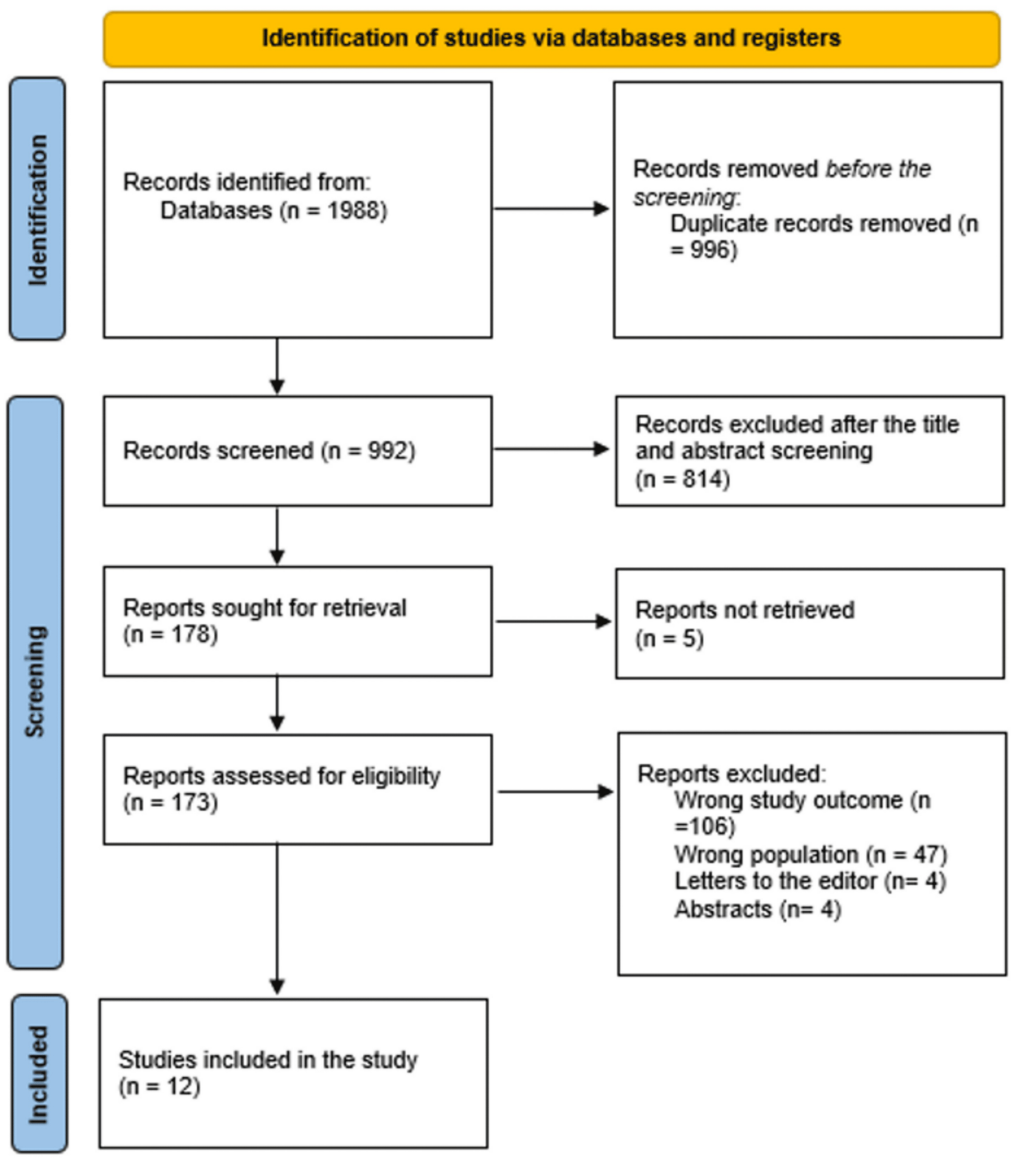
Study decision is summed up in a PRISMA diagram.

After 996 duplicates were removed, a total of 1988 study papers were found through a systematic search. After 992 studies had their titles and abstracts evaluated, 814 papers were discarded. Merely five articles were not located out of the 178 reports that were required to be retrieved. One hundred and seventy-three papers were screened for full-text assessment; 106 were rejected because the study results were wrong, 47 because the population type was inaccurate, 4 articles were editor's letters, and 4 were abstracts. Twelve research publications in this systematic review satisfied the eligibility requirements. An overview of the procedure used to choose the research is illustrated in Figure 1.

Sociodemographic Features of the Comprised Studies

The research publications' sociodemographic information is displayed in Table 1. Twelve studies, including a total of 176,678 patients - 91,447 (51.8%) of them were males - were included in our data [1,12-22]. Five studies were cross-sectional [12,13,16,17,18], four were retrospective cohorts [1,14,15,19], two were prospective cohorts [20,21], and one was case-control [22]. Two studies were conducted in the USA [18,19], two in Turkey [21,22], one in Thailand [12], one in India [13], one in Italy [14], one in Korea [15], one in Saudi Arabia [16], one in Romania [1], one in China [17], and one in Syria [20]. The earliest study was conducted in 2010 [20] and the latest in 2024 [17].

**Table 1:** The sociodemographic attributes of the participating populations.

Study	Study design	Country	Participants	Mean age (years)	Males (%)
Manivannan et al. [12]	Cross-sectional	Thailand	94	4–12	71 (75.5%)
Kumar et al. [13]	Cross-sectional	India	95	5–15	74 (77.9%)
Cantarutti et al. [14]	Retrospective cohort	Italy	86,381	>3	44622 (51.7%)
Kim et al. [15]	Retrospective cohort	Korea	86,096	0–14	44,384 (51.6%)
Alfurayh et al. [16]	Cross-sectional	Saudi Arabia	363	4.9 ± 3.5	229 (63.1%)
Lupu et al. [1]	Retrospective cohort	Romania	56	3–18	41 (73.2%)
Tao et al. [17]	Cross-sectional	China	397	5.7 ± 2.5	252 (63.5%)
Ortega et al. [18]	Cross-sectional	USA	2435	6–12	1356 (56.2%)
Valet et al. [19]	Retrospective cohort	USA	432	<4	235 (54.4%)
Deeb et al. [20]	Prospective cohort	Syria	75	6.89 ± 3.19	55 (73.3%)
Kilic et al. [21]	Prospective cohort	Turkey	50	10.7 ± 2.1	27 (54%)
Özcan et al. [22]	Case-control	Turkey	204	7.8 ± 4.3	101 (49.5%)

Clinical Outcomes

The clinical features are displayed in Table 2. Out of the 12 included studies, only seven reported the method of assessment of GERD. These methods included the International Classification of Diseases-10 (ICD-10) [15], the Boix-Ochoa score [1], the Gastrosoft Programme [21], 24-hour ambulatory intra-esophageal pH monitoring [22], self-reported [18,19], and clinical and endoscopic diagnosis [20]. The prevalence of GERD in asthmatic children ranged from 0.7% [15] to 65.3% [20], with a total prevalence of 3317 (3.6%). The included studies documented that GERD increases the chance of asthma, while asthma raises the risk of GERD [1,12-22]. Obesity in asthmatic patients was an independent risk factor for the incidence of GERD [1]. Controlling asthma is significantly impacted by comorbidities like obesity and GRED [16].

**Table 2:** Clinical features and results of the included research. NM: not mentioned.

Study	Intervention	Management method	Main outcomes	NOS
Manivannan et al. [12]	NM	8 (8.5%)	GERD incidence was higher in obese children.	7
Kumar et al. [13]	NM	44 (46.3%)	The most prevalent concomitant condition is allergic rhinitis, which is followed by GERD, snoring, and psychological distress. Asthma etiology and symptomatology are both impacted by GERD.	7
Cantarutti et al. [14]	NM	1652 (1.9%)	GERD in infancy has been linked to asthma in childhood. Since children with and without treatment for GERD have similar risks, it seems doubtful that acid-suppressive drugs will have a significant impact on the development of asthma.	6
Kim et al. [15]	(ICD)-10	583 (0.7%)	In children, GERD increases the chance of asthma, while asthma raises the risk of GERD.	7
Alfurayh et al. [16]	NM	15 (4.1%)	Controlling asthma is significantly impacted by comorbidities like obesity and GRED.	7
Lupu et al. [1]	Boix-Ochoa score	39 (69.64%)	The statistical test results showed that the presence of asthma boosts the chances of GER by 2.86.	7
Tao et al. [17]	NM	11 (2.7%)	Independent risk factors for SDB in children with asthma include allergic rhinitis, chronic tonsillitis, gastric reflux, adenoid hypertrophy, recurrent respiratory tract infections, and a family history of snoring.	6
Ortega et al. [18]	Self-reported	770 (35.1%)	Exacerbations have been linked to females, Hispanic ethnicity, Medicaid, obesity, sinusitis, GERD, colds, and flu, as well as greater usage of rescue medication.	6
Valet et al. [19]	Self-reported	45 (10.4%)	While having GERD in infancy may increase the severity of an acute respiratory disease, it is not linked to an asthma diagnosis at age 4.	7
Deeb et al. [20]	Clinical and endoscopic	49 (65.3%)	GERD is so common in refractory asthma that all asthmatic patients, regardless of asthma severity, should have it checked. This is especially the case if the patient exhibits any GERD-related symptoms, nocturnal symptoms, or has a negative prick skin test result.	7
Kilic et al. [21]	The Gastrosoft Programme	23 (46%)	The management of atopy, long-acting beta-agonist use, and asthma are not related to the frequency of GERD.	7
Özcan et al. [22]	24-h ambulatory intraoesophageal pH monitoring	78 (38.2%)	The findings indicate that participants who appear with respiratory symptoms indicative of GERD should also be investigated for the presence of underlying asthma, even if patients with and without GERD had equal frequencies of asthma.	7

Discussion

Twelve observational studies that looked at the relationship between childhood GERD and asthma were included in our systematic review. We recorded that the prevalence of GERD in asthmatic children ranged from 0.7% [15] to 65.3% [20], with a total prevalence of 3317 (3.6%). This was lower than Thakkar et al., who reported that the majority of research (n = 19) looked at the prevalence of GERD in 3726 asthmatic people, and the results showed very erratic estimates (19.3-80.0%) and a combined average of 22.8% with GERD symptoms [23].

We also found that the included studies documented that GERD increases the chance of asthma, while asthma raises the risk of GERD [1,12-22]. Obesity in asthmatic patients was an independent risk factor for the incidence of GERD [1]. Controlling asthma is significantly impacted by comorbidities like obesity and GERD [16]. In a review of over 16 studies, including 683 children, Rudolph et al. [24] discovered that over 50% of children with aberrant esophageal pH monitoring studies and recurrent asthma also had no or very mild clinical symptoms of GERD.

A "patient-centred, symptom-based" approach is recommended as the definition of GERD in the paediatric population by a worldwide, evidence-based consensus [25]. Similar prevalence estimates (19.3% and 19.7%) were reported by the only two studies in our analysis that used symptom-based techniques to identify GERD; these values were the lowest of the studies that were included [26,27]. Additionally, vocal descriptions of symptoms may not be reliable until the age of eight, and older children may not be expressive during an office visit [25]. As a result, symptoms-based procedures have substantial limitations when used with children. Furthermore, it might be challenging to identify whether kids genuinely have GERD because childhood symptoms suggestive of the condition are frequently present [28].

The "reflux" and "reflex" theories are the two hypotheses used to explain how GERD affects asthma [29,30]. According to the reflux theory, aspirating stomach contents could cause inflammation in the airways or cause acid to microspirit into the lower airways, which could cause hyperreactivity in the airways. According to the reflex theory, the refluxate causes bronchoconstriction and vagal activation by stimulating receptors in the distal esophagus. The impact of asthma on GERD has been attributed to various mechanisms, including elevated intraabdominal pressure that influences the pressure gradient across the lower esophageal sphincter (LES), altered crural diaphragm-gastric esophageal junction relationship due to hyperinflation, elevated negative intrathoracic pressure from airway obstruction, and asthma therapy that modifies the LES pressure [29,30].

## Conclusions

The findings of our comprehensive review point to a possible link between juvenile patients with asthma who are referred to secondary and tertiary care facilities and having GERD. Nevertheless, the evidence for this link is weak in a number of situations. Lack of longitudinal research establishing the proper temporal sequence, studies indicating no severity-response relationship, and insufficient data showing a treatment-response relationship all contribute to the uncertainty around the nature and direction of the association. Our findings highlight the need for additional epidemiologic research to investigate the connection between GERD and asthma, including long-term follow-up.
